# Aberrant DNA Methylation in Esophageal Squamous Cell Carcinoma: Biological and Clinical Implications

**DOI:** 10.3389/fonc.2020.549850

**Published:** 2020-10-23

**Authors:** Lehang Lin, Xu Cheng, Dong Yin

**Affiliations:** Guangdong Provincial Key Laboratory of Malignant Tumor Epigenetics and Gene Regulation, Medical Research Center, Sun Yat-sen Memorial Hospital, Sun Yat-sen University, Guangzhou, China

**Keywords:** esophageal squamous cell carcinoma, aberrant DNA methylation, global DNA hypomethylation, promoter hypermethylation, heterogeneity, clinical significance

## Abstract

Almost all cancer cells possess multiple epigenetic abnormalities, which cooperate with genetic alterations to enable the acquisition of cancer hallmarks during tumorigenesis. As the most frequently found epigenetic change in human cancers, aberrant DNA methylation manifests at two major forms: global genomic DNA hypomethylation and locus-specific promoter region hypermethylation. It has been recognized as a critical contributor to esophageal squamous cell carcinoma (ESCC) malignant transformation. In ESCC, DNA methylation alterations affect genes involved in cell cycle regulation, DNA damage repair, and cancer-related signaling pathways. Aberrant DNA methylation patterns occur not only in ESCC tumors but also in precursor lesions. It adds another layer of complexity to the ESCC heterogeneity and may serve as early diagnostic, prognostic, and chemo-sensitive markers. Characterization of the DNA methylome in ESCC could help better understand its pathogenesis and develop improved therapies. We herein summarize the current research and knowledge about DNA methylation in ESCC and its clinical significance in diagnosis, prognosis, and treatment.

## Introduction

As the ninth most common cancer in the world, esophageal carcinoma is also the sixth leading cause of cancer-related deaths (1). Esophageal squamous cell carcinoma (ESCC) is the predominant subtype of esophageal carcinoma and accounts for ~90% of the cases (2). Although enormous progress has been made in early diagnosis and multimodal therapies, the prognosis of ESCC patients remains dismal, with the overall 5-year survival rate below 30% (2, 3). To achieve better clinical outcomes, a lot more work needs to be done to understand the pathogenesis of this disease thoroughly.

**Figure 1:**
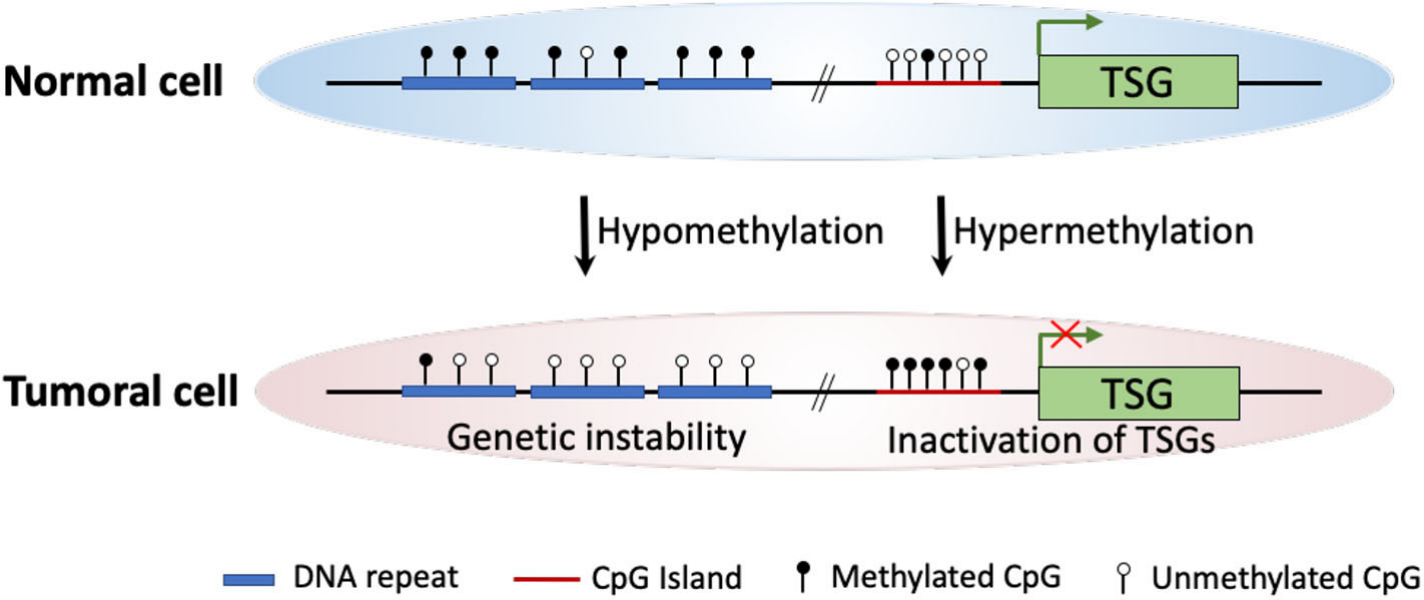
Schematic diagram of major DNA methylation changes in human cancer. In cancer cells, global hypomethylation at repetitive sequences might cause genetic instability, whereas site-specific CpG island promoter hypermethylation could lead to silencing of tumor suppressor genes (TSGs).

Intensive molecular biological studies have revealed that epigenetic dysregulation is a fundamental characteristic of nearly all human cancers (4). The most widely studied epigenetic modification is DNA methylation, which meanwhile is the most frequently found abnormal epigenetic change in human cancers. In mammals, DNA methylation occurs predominantly at the 5′ position of cytosine forming cytosine guanine dinucleotides. This modification is catalyzed and maintained by enzymes known as DNA methyltransferases (DNMTs) (5). The two major DNA methylation changes that occur in human cancers, including ESCC, are global DNA hypomethylation and site-specific CpG island promoter hypermethylation (6, 7). Experimental studies indicate that DNA hypomethylation of repetitive sequences (i.e., long interspersed nucleotide elements, *LINEs*) may predispose cells to chromosomal defects and rearrangements that result in genetic instability. Thus, global DNA hypomethylation increases chromosomal instability, leading to cancer development (8, 9). On the other hand, hypermethylation of the CpG islands located in gene promoter regions may involve in carcinogenesis as a result of three possible mechanisms: (1) cytosine methylation facilitates gene mutation, as 5-methylcytosine is deaminated to thymine; (2) aberrant DNA methylation may be associated with allelic loss; (3) tumor suppressor genes (TSGs) may be inactivated by promoter hypermethylation (7, 10–12) (Figure 1).

In the context of tumor epigenetic heterogeneity, DNA methylation has also been the focus of intense research due to the quantitative nature of DNA methylation assays and the relative ease of obtaining sufficient stable genomic DNA (13, 14). Mechanistically, changes in DNA methylation contribute to the cancer development process through regulation of the spatial chromatin organization and alteration of the transcriptome at the right timing and environment (15). To date, DNA methylation heterogeneity has been quantified and been linked to clinical variables in various cancer types, including prostate cancer (16), chronic lymphocytic leukemia (17), glioblastoma (18), Ewing sarcoma (19), as well as ESCC (20). These findings demonstrate the power of DNA methylation sequencing in analyzing intratumor heterogeneity. At the same time, they suggest the extensive involvement of DNA methylation in tumor development.

In this brief review, we summarize recent advances regarding DNA methylation changes in the initiation and progression of ESCC. We also refer to their possible applications in the clinical management of ESCC patients.

## Global DNA Hypomethylation and Promoter CPG Island Hypermethylation in ESCC

Global DNA hypomethylation in cancer tissues was first observed more than 3 decades ago and has been recognized as a key mechanism involved in carcinogenesis (21, 22). Nevertheless, its presence and importance remain less well-understood in ESCC. To evaluate the global DNA methylation status in ESCC, several studies have measured the methylation level of the *LINE-1*. *LINE-1* constitutes a substantial portion (~17%) of the human genome and has therefore attracted attention as a useful surrogate marker for global DNA methylation (23–27). Importantly, genome-wide hypomethylation was consistently observed in ESCCs, and *LINE-1* hypomethylation was strongly correlated with ESCC progression, leading to a poor prognosis among ESCC patients (23–26). However, this association still awaits further confirmation in larger cohorts, and potential mechanisms by which global DNA hypomethylation affects ESCC behavior have yet to be clearly revealed.

Compared with global genomic DNA hypomethylation, locus-specific DNA hypermethylation, which mostly occurs at promoter CpG islands, has been extensively studied in ESCC by candidate gene approaches. In ESCC, as well as other malignancies, aberrant hypermethylation in promoter CpG islands is involved in the major components of cell cycle regulation, DNA damage repair, and cancer-related signaling pathways. For example, cell cycle-related genes *CDKN2A, RASSF1A*, and *RASSF10* are frequently hypermethylated and transcriptionally silenced in ESCC (28–32). Inactivation of DNA repair genes *MGMT, MLH1*, and *MSH2* in ESCC mainly attributes to the methylation change in their promoter regions (33–35). Promoter hypermethylation in the genes of *APC, RUNX3*, and *ZNF382* respectively affects the Wnt/β-catenin, TGF-β, and NF-κB pathways in ESCC (36–40). Additionally, epigenetic studies on ESCC have discovered many known and putative TSGs that are frequently inactivated by promoter hypermethylation (Table 1). Although growing evidence has demonstrated that tobacco smoking and alcohol consumption are the two major risk factors for ESCC, and correlations between gene promoter methylation and smoking exposure have also been observed in ESCC, the exact triggers and mechanisms responsible for aberrant ESCC DNA methylation changes remain elusive (62–65).

**Table 1:** Aberrantly hypermethylated genes in ESCC.

**Gene**	**Official full name**	**Function**	**References**
*RASSF1*	Ras association domain family member 1	Cell cycle regulation	(24)
*CDKN2A*	Cyclin dependent kinase inhibitor 2A	Apoptosis Modulation and Signaling	(24)
*BRCA1*	BRCA1 DNA Repair Associated	DNA repair	(28)
*CDH1*	Cadherin 1	Cell adhesion	(28)
*RAR-β*	Retinoic acid receptor-beta	Nuclear receptor	(28)
*DAPK*	Death associated protein kinase	Programmed cell death	(28)
*EPHA7*	EPH receptor A7	Brain development	(28, 41)
*MGMT*	O-6-methylguanine-DNA methyltransferase	DNA damage reversal	(33–35)
*MLH1*	MutL homolog 1	DNA mismatch repair	(33, 35)
*MSH2*	MutS homolog 2	DNA mismatch repair	(33, 35)
*APC*	Adenomatous polyposis coli	Wnt signaling pathway	(36)
*RUNX3*	RUNX family transcription factor 3	Notch/TGF-beta receptor signaling pathway	(39)
*ZNF382*	Zinc finger protein 382	Transcription factor	(40)
*FHIT*	Fragile histidine triad diadenosine triphosphatase	Purine metabolism	(42)
*TFF1*	Trefoil factor 1	Secretory protein in gastrointestinal mucosa	(43)
*HIN-1*	Secretoglobin family 3A member (High in normal-1)	Secreted cytokine-like protein	(44)
*ABCB4*	ATP binding cassette subfamily B member 4	ATP-binding cassette transporter	(41)
*PCDH10*	Protocadherin 10	Cell adhesion	(41)
*DOK1*	Docking protein 1	Signal transduction	(41)
*TAC1*	Tachykinin precursor 1	Neurotransmitter	(45)
*DKK3*	Dickkopf WNT signaling pathway inhibitor 3	Wnt signaling modulator	(46)
*SFRP1*	Secreted frizzled related protein 1	Wnt signaling modulator	(47)
*CHL1*	Cell adhesion molecule L1 like	Signal transduction	(48)
*RHCG*	Rh family C glycoprotein	Ammonium transporter	(49)
*PAX5*	Paired box 5	Neural development; spermatogenesis	(50, 51)
*CHFR*	Checkpoint with forkhead and ring finger domains	Cell cycle progression	(52)
*TFPI2*	Tissue factor pathway inhibitor 2	Cell adhesion; plasmin signaling	(53)
*BIN1*	Bridging integrator 1	Neuroscience	(54)
*ZNF132*	Zinc finger protein 132	Transcription factor	(55)
*PTX3*	Pentraxin 3	Innate immune system; inflammatory reactions	(56)
*RAB25*	RAB25, member RAS oncogene family	Membrane trafficking	(57)
*ECRG4*	ECRG4 augurin precursor	Senescence	(58)
*SPINT2*	Serine peptidase inhibitor, Kunitz type 2	Transmembrane protein	(59)
*EYA4*	EYA transcriptional coactivator and phosphatase 4	DNA repair	(60)
*SEMA3B*	Semaphorin 3B	Neuronal development	(61)

## DNA Methylation Change is a Critical Event in the Pathogenesis of ESCC

The histologic progression of ESCC is a multi-step process that begins from normal squamous epithelium to low-grade dysplasia, high-grade dysplasia, carcinoma *in situ*, and invasive carcinoma (66, 67). In addition to genetic alterations, epigenetic abnormalities, in particular DNA methylation changes, have become widely accepted as common molecular features during ESCC development (68–70).

There is growing recognition that aberrant DNA methylation patterns occur in the earliest stage of ESCC and may parallel the histologic changes observed during ESCC carcinogenesis. For example, promoter methylation of TSGs, including *p16, p14, FHIT, MGMT*, and *TFF1* was detected in precancerous dysplastic lesions, indicating their early development in ESCC (42, 43). High in normal-1 (*HIN-1*) is a tumor suppressor gene that is highly expressed in many epithelial tissues. Methylation of this gene promoter occurs in the early stages of ESCC and accumulates with esophageal progression tendency during carcinogenesis, resulting in *HIN-1* silencing in carcinomas-*in*-*situ* (44). By analyzing normal esophageal mucosa, esophageal dysplasia, and ESCC, Guo et al. also found clear evidence of accumulated methylation events during ESCC progression, which included accumulation of promoter region methylation of genes *MGMT, p16, BRCA1, MLH1, CDH1, RAR*β*2, DAPK*, and *APC* (28, 71). These findings are reminiscent of the idea that hypermethylation abnormalities may represent a “field cancerization” or “field defect” of preneoplastic changes, which occur early in carcinogenesis and predispose cells to subsequent malignant transformation (72–74).

From the standpoint of epigenetic heterogeneity, DNA methylation change is also involved in the evolutionary trajectory of ESCC. In 1976, Peter C. Nowell firstly proposed the Darwinian-like clonal evolution of tumor cells (75). Since then, models of tumor evolution derived from intratumor heterogeneity have greatly improved our understanding of tumorigenesis (76, 77). Investigations of intratumor heterogeneity at the DNA methylation level have provided unique opportunities in identifying epigenetic alterations associated with tumor evolution.

In ESCC, DNA methylation status within promoters of transcription factors SIM2 and SIX1 is strongly correlated with their heterogeneous expression pattern, which is further associated with ESCC differentiation and progression (78–80). Using the Illumina Human Methylation 450K (HM450) Bead array, Hao et al. performed multi-region global methylation profiling on 3 ESCC patients and found numbers of TSGs, including *EPHA7, ABCB4, PCDH10, DOK1*, etc., were heterogeneously hypermethylated at their promoters within tumors from the same individual (41). This observation suggests that intratumor heterogeneous DNA methylation may play a role in the subclonal diversification of ESCC tumors. Moreover, the construction of phylogenies independently from DNA methylation (phyloepigenetic tree) and somatic mutations (phylogenetic tree) yielded highly concordant as well as complementary evolutionary histories (41). This finding supports the concept of co-dependency of aberrant DNA methylation and genetic alterations and again demonstrates the involvement of DNA methylation heterogeneity in the ESCC evolutionary process. In an attempt to gain deeper insights into the DNA methylation heterogeneity of ESCC, most recently, Teng et al. generated single-base resolution whole-genome bisulfite sequencing data on 84 ESCCs and paired paraneoplastic tissues (20). Their analysis also identified numerous DNA methylation alterations associated with ESCC carcinogenesis and lymph node metastasis. In general, they found ESCC manifested substantial inter- and intratumor DNA methylation heterogeneity. Strikingly, the degree of intertumor DNA methylation heterogeneity of ESCC was even higher than that of prostate cancer or chronic lymphocytic leukemia, two well-recognized heterogeneous cancer types (16, 17). Furthermore, ESCC patients with a great extent of DNA methylation heterogeneity tended to experience more aggressive disease and worse overall survival (20).

Altogether, these data suggest the biological significance of DNA methylation change in ESCC. However, additional research is required to determine the extent to which intratumor heterogeneity of DNA methylation reflects differences in regional or clone-specific driver or passenger events of ESCC, and to what extent the alterations to DNA methylation and genetics may be functionally related. It is also not yet known whether and how the pattern of DNA methylation change will evolve during ESCC progression.

## Clinical Implications of DNA Methylation Change in ESCC

In several cancer types, the methods of detecting aberrant DNA methylation changes have been applied to clinical fields related to early diagnosis, prognostic prediction, and personalized therapy.

In ESCC, as with other cancers, early detection is a prerequisite for treatment success and survival improvement. In fact, aberrantly methylated genes have been detected in the plasma or serum from ESCC patients, implying that they may serve as non-invasive diagnostic biomarkers. Using methylation-specific polymerase chain reaction, Das et al. detected the methylation status of *MGMT* from 100 newly diagnosed ESCC patients and reported that promoter hypermethylation of *MGMT* was present in 70% of serum samples (81). Zheng et al. evaluated the diagnostic role of *RUNX3* methylation in serum DNA of ESCCs and found that *RUNX3* hypermethylation was detectable in 51.4% (36/70) of the cases, which was significantly higher than that of the healthy donors (39). Moreover, Li et al. showed that the serum DNA detection of a panel of methylated genes, including *RAR-*β, *DAPK, CDH1, p16*, and *RASSF1A*, had a high diagnostic sensitivity of 82% and specificity of 100% for ESCC patients. They thereby suggested that it might be more efficient to early diagnose ESCC through integrative analysis of the methylation status of multiple TSGs (33). Of note, the early diagnostic value of aberrant methylation changes in serum DNA has been widely investigated by many other studies in ESCC. Such methylation events include hypermethylation of genes *SFRP1, CASZ1, CDH13, ING2, DKK-3, TAC1*, etc. (45–47, 82).

The aberrant methylation pattern has also been identified as a prognostic indicator for ESCC patients. Recently, promoter hypermethylation of *CHL1* and *RHCG*, two novel tumor suppressor candidates, has been reported to be associated with poor differentiation and increased invasion of ESCC, as well as advanced tumor stage and decreased overall survival (48, 49). Using DNA from the plasma of ESCC patients, Liu et al. analyzed the methylation status of Wnt antagonists *SFRP1, DKK3*, and *RUNX3* and showed that patients carrying two or three of these hypermethylated genes had a significantly elevated risk of cancer recurrence, compared with those without methylated genes (46). In an attempt to identify DNA methylation markers in predicting lymph node metastasis, the genome-wide methylation of 86 ESCC patients has been recently assessed, and a 10-probe lymph node metastasis-associated methylation signature has been established through stringent bioinformatics analyses (83). All in all, finding genes of prognostic impact aberrantly methylated allows one to customize therapy for ESCC patients: patients with a worse prognosis might benefit from a more aggressive treatment strategy, while patients with low risk can forego radical surgery.

Methylation might result in the deactivation of genes that are responsive or unresponsive to chemotherapy and radiotherapy. Therefore, the methylation status of certain genes can be used to predict a patient's treatment response in advance (84). The best known example is *MGMT* promoter methylation and the resultant response to DNA alkylating agents in gliomas (85). However, ESCC studies in this regard are very limited. *PAX5* gene methylation was identified as an excellent marker for squamous cell carcinoma detection (50). According to Kurimoto et al.'s study, hypermethylation of *PAX5* was significantly correlated with increased ESCC cell proliferation and cisplatin resistance, leading to poor recurrence-free survival and overall survival (51). *CHFR* is an early mitotic checkpoint gene that controls cell cycle progression at the G2/M checkpoint and maintains chromosomal integrity (86). Promoter region methylation of *CHFR* was found frequently in ESCC and shown to sensitize ESCC to taxane treatment (52).

## Discussion

Epigenetic alterations have been recognized as key contributors to cancer initiation and progression. Amongst these, DNA methylation is one of the most extensively studied epigenetic modifications that occurs in the earliest stage of cancers (87). The aim of this review is to discuss the aberrant DNA methylation changes affecting ESCC carcinogenesis. However, many investigations into such epigenomic features of ESCC are still in their infancy.

Depending on the genomic location, DNA methylation may have different biological functions. Methylation in promoter CpG islands is typically associated with gene repression, while methylation in the gene body is usually linked to active gene expression. Accumulating evidence has shown that intergenic regions contain many regulatory elements, such as enhancers, silencers, and non-coding RNAs, and their functions may also be affected by DNA methylation (84). However, there is a current lack of knowledge with regards to these types of changes during ESCC development. Thus, the ESCC methylome landscape awaits further characterization through sequence-based approaches such as whole-genome bisulfite sequencing at single-base levels of resolution.

DNA methylation changes have emerged as clinically-relevant disease markers due to their chemical stability and measurable feasibility (88). As mentioned previously, there are numerous candidate methylation events that may serve as diagnostic, prognostic, and chemo-sensitive markers in ESCC. However, the cancer type-specificity of many of these methylation changes remains to be determined in larger ESCC cohorts. Their predictive and prognostic value is also challenged by the property of tumor heterogeneity, especially when phenotypically diversified cancerous cells are unequally distributed and asynchronously evolving over space and time. This entails personalized and longitudinal studies assessing methylation changes in the course of ESCC development. It is also worth investigating whether the extent of DNA methylation heterogeneity by itself could be used as a clinically useful biomarker in ESCC.

Unlike genetic alterations that are essentially fixed, epigenetic changes, including DNA hypermethylation, are intriguingly dynamic and theoretically reversible. This makes them attractive targets for cancer therapy or chemoprevention (89). Indeed, several studies have demonstrated that TSG expression could be restored after treatment of cells with demethylating agents, and DNMT inhibitors, such as azacytidine and decitabine, are currently under preclinical and clinical investigations in various cancer types (e.g., ClinicalTrials.gov Identifier: NCT01193517, NCT03666559, and NCT04187703) (90–93). Nonetheless, up until now, very limited data have been shown concerning the effectiveness of DNMT inhibitors in ESCC. Future research on this field are required to gain a more in-depth insight into ESCC development and open a new area for ESCC treatment. Ideally, we will finally reach our goal of “epigenetic precision medicine” in ESCC.

## Author Contributions

LL conceived and wrote the review. XC and DY helped the revision. All authors contributed to the article and approved the submitted version.

## Conflict of Interest

The authors declare that the research was conducted in the absence of any commercial or financial relationships that could be construed as a potential conflict of interest. The handling editor declared a shared affiliation, though no other collaboration, with the authors.

## References

[1] Bray F Ferlay J Soerjomataram I Siegel RL Torre LA Jemal A. Global cancer statistics 2018: GLOBOCAN estimates of incidence and mortality worldwide for 36 cancers in 185 countries. CA Cancer J Clin. (2018) 68:394–424. 10.3322/caac.21492 30207593

[2] Pennathur A Gibson MK Jobe BA Luketich JD. Oesophageal carcinoma. Lancet. (2013) 381:400–12. 10.1016/S0140-6736(12)60643-6 23374478

[3] Holmes RS Vaughan TL. Epidemiology and pathogenesis of esophageal cancer. Semin Radiat Oncol. (2007) 17:2–9. 10.1016/j.semradonc.2006.09.003 17185192

[4] Jones PA Baylin SB The epigenomics of cancer. Cell. (2007) 128:683–92. 10.1016/j.cell.2007.01.029 17320506 PMC3894624

[5] Laird PW. The power and the promise of DNA methylation markers. Nat Rev Cancer. (2003) 3:253–66. 10.1038/nrc1045 12671664

[6] Ahrens TD Werner M Lassmann S. Epigenetics in esophageal cancers. Cell Tissue Res. (2014) 356:643–55. 10.1007/s00441-014-1876-y 24816987

[7] Baba Y Watanabe M Baba H. Review of the alterations in DNA methylation in esophageal squamous cell carcinoma. Surg Today. (2013) 43:1355–64. 10.1007/s00595-012-0451-y 23291904

[8] Ehrlich M. DNA hypomethylation in cancer cells. Epigenomics. (2009) 1:239–59. 10.2217/epi.09.33 20495664 PMC2873040

[9] Eden A Gaudet F Waghmare A Jaenisch R Chromosomal instability and tumors promoted by DNA hypomethylation. Science. (2003) 300:455 10.1126/science.1083557 12702868

[10] Shen JC Rideout WM III Jones PA. High frequency mutagenesis by a DNA methyltransferase. Cell. (1992) 71:1073–80. 10.1016/S0092-8674(05)80057-1 1473145

[11] Robertson KD DNA methylation, methyltransferases, and cancer. Oncogene. (2001) 20:3139–55. 10.1038/sj.onc.1204341 11420731

[12] Jones PA Laird PW. Cancer epigenetics comes of age. Nat Genet. (1999) 21:163–7. 10.1038/5947 9988266

[13] Mazor T Pankov A Song JS Costello JF. Intratumoral heterogeneity of the epigenome. Cancer Cell. (2016) 29:440–51. 10.1016/j.ccell.2016.03.009 27070699 PMC4852161

[14] Marusyk A Almendro V Polyak K. Intra-tumour heterogeneity: a looking glass for cancer? Nat Rev Cancer. (2012) 12:323–34. 10.1038/nrc3261 22513401

[15] Liu J Dang H Wang XW. The significance of intertumor and intratumor heterogeneity in liver cancer. Exp Mol Med. (2018) 50:e416. 10.1038/emm.2017.165 29303512 PMC5992990

[16] Fraser M Sabelnykova VY Yamaguchi TN Heisler LE Livingstone J Huang V. Genomic hallmarks of localized, non-indolent prostate cancer. Nature. (2017) 541:359–64. 10.1038/nature20788 28068672

[17] Landau DA Clement K Ziller MJ Boyle P Fan J Gu H. Locally disordered methylation forms the basis of intratumor methylome variation in chronic lymphocytic leukemia. Cancer Cell. (2014) 26:813–25. 10.1016/j.ccell.2014.10.012 25490447 PMC4302418

[18] Klughammer J Kiesel B Roetzer T Fortelny N Nemc A Nenning KH. The DNA methylation landscape of glioblastoma disease progression shows extensive heterogeneity in time and space. Nat Med. (2018) 24:1611–24. 10.1038/s41591-018-0156-x 30150718 PMC6181207

[19] Sheffield NC Pierron G Klughammer J Datlinger P Schonegger A Schuster M. DNA methylation heterogeneity defines a disease spectrum in Ewing sarcoma. Nat Med. (2017) 23:386–95. 10.1038/nm.4273 28134926 PMC5951283

[20] Teng H Xue M Liang J Wang X Wang L Wei W. Inter- and intratumor DNA methylation heterogeneity associated with lymph node metastasis and prognosis of esophageal squamous cell carcinoma. Theranostics. (2020) 10:3035–48. 10.7150/thno.42559 32194853 PMC7053185

[21] Feinberg AP Vogelstein B. Hypomethylation distinguishes genes of some human cancers from their normal counterparts. Nature. (1983) 301:89–92. 10.1038/301089a0 6185846

[22] Gaudet F Hodgson JG Eden A Jackson-Grusby L Dausman J Gray JW. Induction of tumors in mice by genomic hypomethylation. Science. (2003) 300:489–92. 10.1126/science.1083558 12702876

[23] Kawano H Saeki H Kitao H Tsuda Y Otsu H Ando K. Chromosomal instability associated with global DNA hypomethylation is associated with the initiation and progression of esophageal squamous cell carcinoma. Ann Surg Oncol. (2014) 21:S696–702. 10.1245/s10434-014-3818-z 24898425

[24] Hoshimoto S Takeuchi H Ono S Sim MS Huynh JL Huang SK. Genome-wide hypomethylation and specific tumor-related gene hypermethylation are associated with esophageal squamous cell carcinoma outcome. J Thorac Oncol. (2015) 10:509–17. 10.1097/JTO.0000000000000441 25514805

[25] Baba Y Watanabe M Murata A Shigaki H Miyake K Ishimoto T. LINE-1 hypomethylation, DNA copy number alterations, and CDK6 amplification in esophageal squamous cell carcinoma. Clin Cancer Res. (2014) 20:1114–24. 10.1158/1078-0432.CCR-13-1645 24423610

[26] Iwagami S Baba Y Watanabe M Shigaki H Miyake K Ishimoto T. LINE-1 hypomethylation is associated with a poor prognosis among patients with curatively resected esophageal squamous cell carcinoma. Ann Surg. (2013) 257:449–55. 10.1097/SLA.0b013e31826d8602 23023202

[27] Cordaux R Batzer MA. The impact of retrotransposons on human genome evolution. Nat Rev Genet. (2009) 10:691–703. 10.1038/nrg2640 19763152 PMC2884099

[28] Guo M Ren J House MG Qi Y Brock MV Herman JG. Accumulation of promoter methylation suggests epigenetic progression in squamous cell carcinoma of the esophagus. Clin Cancer Res. (2006) 12:4515–22. 10.1158/1078-0432.CCR-05-2858 16899597

[29] Du Z Ma K Sun X Li A Wang H Zhang L. Methylation of RASSF1A gene promoter and the correlation with DNMT1 expression that may contribute to esophageal squamous cell carcinoma. World J Surg Oncol. (2015) 13:141. 10.1186/s12957-015-0557-y 25886188 PMC4403718

[30] Kuroki T Trapasso F Yendamuri S Matsuyama A Alder H Mori M. Promoter hypermethylation of RASSF1A in esophageal squamous cell carcinoma. Clin Cancer Res. (2003) 9:1441–5. 12684417

[31] Mao WM Li P Zheng QQ Wang CC Ge MH Hu FJ. Hypermethylation-modulated downregulation of RASSF1A expression is associated with the progression of esophageal cancer. Arch Med Res. (2011) 42:182–8. 10.1016/j.arcmed.2011.04.002 21722812

[32] Lu D Ma J Zhan Q Li Y Qin J Guo M. Epigenetic silencing of RASSF10 promotes tumor growth in esophageal squamous cell carcinoma. Discov Med. (2014) 17:169–78. 24759621

[33] Ling ZQ Li P Ge MH Hu FJ Fang XH Dong ZM. Aberrant methylation of different DNA repair genes demonstrates distinct prognostic value for esophageal cancer. Dig Dis Sci. (2011) 56:2992–3004. 10.1007/s10620-011-1774-z 21674174

[34] Fang MZ Jin Z Wang Y Liao J Yang GY Wang LD. Promoter hypermethylation and inactivation of O(6)-methylguanine-DNA methyltransferase in esophageal squamous cell carcinomas and its reactivation in cell lines. Int J Oncol. (2005) 26:615–22. 10.3892/ijo.26.3.615 15703815

[35] Su Y Yin L Liu R Sheng J Yang M Wang Y. Promoter methylation status of MGMT, hMSH2, and hMLH1 and its relationship to corresponding protein expression and TP53 mutations in human esophageal squamous cell carcinoma. Med Oncol. (2014) 31:784. 10.1007/s12032-013-0784-4 24366688

[36] Zare M Jazii FR Alivand MR Nasseri NK Malekzadeh R Yazdanbod M. Qualitative analysis of adenomatous polyposis coli promoter: hypermethylation, engagement and effects on survival of patients with esophageal cancer in a high risk region of the world, a potential molecular marker. BMC Cancer. (2009) 9:24. 10.1186/1471-2407-9-24 19149902 PMC2637891

[37] Kim YT Park JY Jeon YK Park SJ Song JY Kang CH. Aberrant promoter CpG island hypermethylation of the adenomatosis polyposis coli gene can serve as a good prognostic factor by affecting lymph node metastasis in squamous cell carcinoma of the esophagus. Dis Esophagus. (2009) 22:143–50. 10.1111/j.1442-2050.2008.00862.x 18847451

[38] Ishii T Murakami J Notohara K Cullings HM Sasamoto H Kambara T. Oesophageal squamous cell carcinoma may develop within a background of accumulating DNA methylation in normal and dysplastic mucosa. Gut. (2007) 56:13–9. 10.1136/gut.2005.089813 16785283 PMC1856655

[39] Zheng Y Zhang Y Huang X Chen L. Analysis of the RUNX3 gene methylation in serum DNA from esophagus squamous cell carcinoma, gastric and colorectal adenocarcinoma patients. Hepatogastroenterology. (2011) 58:2007–11. 10.5754/hge10016 22234069

[40] Cheng Y Geng H Cheng SH Liang P Bai Y Li J. KRAB zinc finger protein ZNF382 is a proapoptotic tumor suppressor that represses multiple oncogenes and is commonly silenced in multiple carcinomas. Cancer Res. (2010) 70:6516–26. 10.1158/0008-5472.CAN-09-4566 20682794

[41] Hao JJ Lin DC Dinh HQ Mayakonda A Jiang YY Chang C. Spatial intratumoral heterogeneity and temporal clonal evolution in esophageal squamous cell carcinoma. Nat Genet. (2016) 48:1500–7. 10.1038/ng.3683 27749841 PMC5127772

[42] Li JS Ying JM Wang XW Wang ZH Tao Q Li LL. Promoter methylation of tumor suppressor genes in esophageal squamous cell carcinoma. Chin J Cancer. (2013) 32:3–11. 10.5732/cjc.011.10381 22572016 PMC3845589

[43] Lima SC Hernandez-Vargas H Simao T Durand G Kruel CD Le Calvez-Kelm F. Identification of a DNA methylome signature of esophageal squamous cell carcinoma and potential epigenetic biomarkers. Epigenetics. (2011) 6:1217–27. 10.4161/epi.6.10.17199 21946330

[44] Guo M Ren J Brock MV Herman JG Carraway HE. Promoter methylation of HIN-1 in the progression to esophageal squamous cancer. Epigenetics. (2008) 3:336–41. 10.4161/epi.3.6.7158 19098448

[45] Jin Z Olaru A Yang J Sato F Cheng Y Kan T. Hypermethylation of tachykinin-1 is a potential biomarker in human esophageal cancer. Clin Cancer Res. (2007) 13:6293–300. 10.1158/1078-0432.CCR-07-0818 17975140

[46] Liu JB Qiang FL Dong J Cai J Zhou SH Shi MX. Plasma DNA methylation of Wnt antagonists predicts recurrence of esophageal squamous cell carcinoma. World J Gastroenterol. (2011) 17:4917–21. 10.3748/wjg.v17.i44.4917 22171134 PMC3235636

[47] Liu C Li N Lu H Wang Z Chen C Wu L. Circulating SFRP1 promoter methylation status in gastric adenocarcinoma and esophageal square cell carcinoma. Biomed Rep. (2015) 3:123–7. 10.3892/br.2014.388 25469261 PMC4251162

[48] Tang H Jiang L Zhu C Liu R Wu Y Yan Q. Loss of cell adhesion molecule L1 like promotes tumor growth and metastasis in esophageal squamous cell carcinoma. Oncogene. (2019) 38:3119–33. 10.1038/s41388-018-0648-7 30622339

[49] Ming XY Zhang X Cao TT Zhang LY Qi JL Kam NW. RHCG suppresses tumorigenicity and metastasis in esophageal squamous cell carcinoma via inhibiting NF-kappaB signaling and MMP1 expression. Theranostics. (2018) 8:185–98. 10.7150/thno.21383 29290801 PMC5743468

[50] Guerrero-Preston R Michailidi C Marchionni L Pickering CR Frederick MJ Myers JN. Key tumor suppressor genes inactivated by “greater promoter” methylation and somatic mutations in head and neck cancer. Epigenetics. (2014) 9:1031–46. 10.4161/epi.29025 24786473 PMC4143405

[51] Kurimoto K Hayashi M Guerrero-Preston R Koike M Kanda M Hirabayashi S. PAX5 gene as a novel methylation marker that predicts both clinical outcome and cisplatin sensitivity in esophageal squamous cell carcinoma. Epigenetics. (2017) 12:865–74. 10.1080/15592294.2017.1365207 29099287 PMC5788430

[52] Yun T Liu Y Gao D Linghu E Brock MV Yin D. Methylation of CHFR sensitizes esophageal squamous cell cancer to docetaxel and paclitaxel. Genes Cancer. (2015) 6:38–48. 10.18632/genesandcancer.46 25821560 PMC4362483

[53] Wang C Pu W Zhao D Zhou Y Lu T Chen S. Identification of hyper-methylated tumor suppressor genes-based diagnostic panel for esophageal squamous cell carcinoma (ESCC) in a Chinese han population. Front Genet. (2018) 9:356. 10.3389/fgene.2018.00356 30233644 PMC6133993

[54] Wang X Wang J Jia Y Wang Y Han X Duan Y. Methylation decreases the Bin1 tumor suppressor in ESCC and restoration by decitabine inhibits the epithelial mesenchymal transition. Oncotarget. (2017) 8:19661–73. 10.18632/oncotarget.14914 28152502 PMC5386712

[55] Jiang D He Z Wang C Zhou Y Li F Pu W. Epigenetic silencing of ZNF132 mediated by methylation-sensitive Sp1 binding promotes cancer progression in esophageal squamous cell carcinoma. Cell Death Dis. (2018) 10:1. 10.1038/s41419-018-1236-z 30578410 PMC6315024

[56] Wang JX He YL Zhu ST Yang S Zhang ST. Aberrant methylation of the 3q25 tumor suppressor gene PTX3 in human esophageal squamous cell carcinoma. World J Gastroenterol. (2011) 17:4225–30. 10.3748/wjg.v17.i37.4225 22072855 PMC3208368

[57] Tong M Chan KW Bao JY Wong KY Chen JN Kwan PS. Rab25 is a tumor suppressor gene with antiangiogenic and anti-invasive activities in esophageal squamous cell carcinoma. Cancer Res. (2012) 72:6024–35. 10.1158/0008-5472.CAN-12-1269 22991305

[58] Li LW Yu XY Yang Y Zhang CP Guo LP Lu SH. Expression of esophageal cancer related gene 4 (ECRG4), a novel tumor suppressor gene, in esophageal cancer and its inhibitory effect on the tumor growth *in vitro* and *in vivo*. Int J Cancer. (2009) 125:1505–13. 10.1002/ijc.24513 19521989

[59] Yue D Fan Q Chen X Li F Wang L Huang L. Epigenetic inactivation of SPINT2 is associated with tumor suppressive function in esophageal squamous cell carcinoma. Exp Cell Res. (2014) 322:149–58. 10.1016/j.yexcr.2013.11.009 24269829

[60] Luo M Li Y Shi X Yang W Zhou F Sun N. Aberrant methylation of EYA4 promotes epithelial-mesenchymal transition in esophageal squamous cell carcinoma. Cancer Sci. (2018) 109:1811–24. 10.1111/cas.13615 29660222 PMC5989845

[61] Dong Z Liang X Wu X Kang X Guo Y Shen S. Promoter hypermethylation-mediated downregulation of tumor suppressor gene SEMA3B and lncRNA SEMA3B-AS1 correlates with progression and prognosis of esophageal squamous cell carcinoma. Clin Exp Metastasis. (2019) 36:225–41. 10.1007/s10585-019-09964-3 30915595

[62] Tanaka F Yamamoto K Suzuki S Inoue H Tsurumaru M Kajiyama Y Strong interaction between the effects of alcohol consumption and smoking on oesophageal squamous cell carcinoma among individuals with ADH1B and/or ALDH2 risk alleles. Gut. (2010) 59:1457–64. 10.1136/gut.2009.205724 20833657

[63] Anantharaman D Marron M Lagiou P Samoli E Ahrens W Pohlabeln H. Population attributable risk of tobacco and alcohol for upper aerodigestive tract cancer. Oral Oncol. (2011) 47:725–31. 10.1016/j.oraloncology.2011.05.004 21684805

[64] Pelucchi C Gallus S Garavello W Bosetti C La Vecchia C. Cancer risk associated with alcohol and tobacco use: focus on upper aero-digestive tract and liver. Alcohol Res Health. (2006) 29:193–8. 17373408 PMC6527045

[65] Huang Y Chang X Lee J Cho YG Zhong X Park IS. Cigarette smoke induces promoter methylation of single-stranded DNA-binding protein 2 in human esophageal squamous cell carcinoma. Int J Cancer. (2011) 128:2261–73. 10.1002/ijc.25569 20658532 PMC3206631

[66] Lin L Lin DC. Biological significance of tumor heterogeneity in esophageal squamous cell carcinoma. Cancers. (2019) 11:1156. 10.3390/cancers11081156 31409002 PMC6721624

[67] Lin DC Wang MR Koeffler HP. Genomic and epigenomic aberrations in esophageal squamous cell carcinoma and implications for patients. Gastroenterology. (2018) 154:374–89. 10.1053/j.gastro.2017.06.066 28757263 PMC5951382

[68] Toh Y Egashira A Yamamoto M. Epigenetic alterations and their clinical implications in esophageal squamous cell carcinoma. Gen Thorac Cardiovasc Surg. (2013) 61:262–9. 10.1007/s11748-013-0235-3 23529258

[69] You JS Jones PA. Cancer genetics and epigenetics: two sides of the same coin? Cancer Cell. (2012) 22:9–20. 10.1016/j.ccr.2012.06.008 22789535 PMC3396881

[70] Sawan C Vaissiere T Murr R Herceg Z. Epigenetic drivers and genetic passengers on the road to cancer. Mutat Res. (2008) 642:1–13. 10.1016/j.mrfmmm.2008.03.002 18471836

[71] Vogelstein B Kinzler KW. The multistep nature of cancer. Trends Genet. (1993) 9:138–41. 10.1016/0168-9525(93)90209-Z 8516849

[72] Guo M House MG Hooker C Han Y Heath E Gabrielson E. Promoter hypermethylation of resected bronchial margins: a field defect of changes? Clin Cancer Res. (2004) 10:5131–6. 10.1158/1078-0432.CCR-03-0763 15297416

[73] Slaughter DP Southwick HW Smejkal W. Field cancerization in oral stratified squamous epithelium; clinical implications of multicentric origin. Cancer. (1953) 6:963–8. 10.1002/1097-0142(195309)6:5<963::aid-cncr2820060515>3.0.co;2-q 13094644

[74] Leedham SJ Graham TA Oukrif D McDonald SA Rodriguez-Justo M Harrison RF. Clonality, founder mutations, and field cancerization in human ulcerative colitis-associated neoplasia. Gastroenterology. (2009) 136:542–50.e6. 10.1053/j.gastro.2008.10.086 19103203

[75] Nowell PC. The clonal evolution of tumor cell populations. Science. (1976) 194:23–8. 10.1126/science.959840 959840

[76] Swanton C. Intratumor heterogeneity: evolution through space and time. Cancer Res. (2012) 72:4875–82. 10.1158/0008-5472.CAN-12-2217 23002210 PMC3712191

[77] Gerlinger M Rowan AJ Horswell S Math M Larkin J Endesfelder D. Intratumor heterogeneity and branched evolution revealed by multiregion sequencing. N Engl J Med. (2012) 366:883–92. 10.1056/NEJMoa1113205 22397650 PMC4878653

[78] Komatsu M Sasaki H. DNA methylation is a key factor in understanding differentiation phenotype in esophageal squamous cell carcinoma. Epigenomics. (2014) 6:567–9. 10.2217/epi.14.56 25531249

[79] Nishimura T Tamaoki M Komatsuzaki R Oue N Taniguchi H Komatsu M. SIX1 maintains tumor basal cells via transforming growth factor-beta pathway and associates with poor prognosis in esophageal cancer. Cancer Sci. (2017) 108:216–25. 10.1111/cas.13135 27987372 PMC5329162

[80] Tamaoki M Komatsuzaki R Komatsu M Minashi K Aoyagi K Nishimura T. Multiple roles of single-minded 2 in esophageal squamous cell carcinoma and its clinical implications. Cancer Sci. (2018) 109:1121–34. 10.1111/cas.13531 29427302 PMC5891185

[81] Das M Sharma SK Sekhon GS Saikia BJ Mahanta J Phukan RK. Promoter methylation of MGMT gene in serum of patients with esophageal squamous cell carcinoma in North East India. Asian Pac J Cancer Prev. (2014) 15:9955–60. 10.7314/APJCP.2014.15.22.9955 25520135

[82] Wang HQ Yang CY Wang SY Wang T Han JL Wei K. Cell-free plasma hypermethylated CASZ1, CDH13 and ING2 are promising biomarkers of esophageal cancer. J Biomed Res. (2018) 32:424–33. 10.7555/JBR.32.20170065 30355852 PMC6283827

[83] Roy R Kandimalla R Sonohara F Koike M Kodera Y Takahashi N. A comprehensive methylation signature identifies lymph node metastasis in esophageal squamous cell carcinoma. Int J Cancer. (2019) 144:1160–9. 10.1002/ijc.31755 30006931 PMC7331270

[84] Yan W Herman JG Guo M. Epigenome-based personalized medicine in human cancer. Epigenomics. (2016) 8:119–33. 10.2217/epi.15.84 26344672

[85] Esteller M Garcia-Foncillas J Andion E Goodman SN Hidalgo OF Vanaclocha V. Inactivation of the DNA-repair gene MGMT and the clinical response of gliomas to alkylating agents. N Engl J Med. (2000) 343:1350–4. 10.1056/NEJM200011093431901 11070098

[86] Scolnick DM Halazonetis TD. Chfr defines a mitotic stress checkpoint that delays entry into metaphase. Nature. (2000) 406:430–5. 10.1038/35019108 10935642

[87] Kulis M Esteller M. DNA methylation and cancer. Adv Genet. (2010) 70:27–56. 10.1016/B978-0-12-380866-0.60002-2 20920744

[88] Guo M Peng Y Gao A Du C Herman JG Epigenetic heterogeneity in cancer. Biomark Res. (2019) 7:23 10.1186/s40364-019-0174-y 31695915 PMC6824025

[89] Feinberg AP. The key role of epigenetics in human disease prevention and mitigation. N Engl J Med. (2018) 378:1323–34. 10.1056/NEJMra1402513 29617578 PMC11567374

[90] Schrump DS Fischette MR Nguyen DM Zhao M Li X Kunst TF Phase I study of decitabine-mediated gene expression in patients with cancers involving the lungs, esophagus, or pleura. Clin Cancer Res. (2006) 12:5777–85. 10.1158/1078-0432.CCR-06-0669 17020984

[91] Martin I Navarro B Solano C Calabuig M Hernandez-Boluda JC Amat P. Synergistic antioncogenic activity of azacitidine and curcumin in myeloid leukemia cell lines and patient samples. Anticancer Res. (2019) 39:4757–66. 10.21873/anticanres.13659 31519576

[92] Giri AK Aittokallio T. DNMT inhibitors increase methylation in the cancer genome. Front Pharmacol. (2019) 10:385. 10.3389/fphar.2019.00385 31068808 PMC6491738

[93] Moufarrij S Dandapani M Arthofer E Gomez S Srivastava A Lopez-Acevedo M. Epigenetic therapy for ovarian cancer: promise and progress. Clin Epigenetics. (2019) 11:7. 10.1186/s13148-018-0602-0 30646939 PMC6334391

